# Differences in integrin expression and signaling within human breast cancer cells

**DOI:** 10.1186/1471-2407-11-293

**Published:** 2011-07-13

**Authors:** Aliakbar Taherian, Xinlei Li, Yongqing Liu, Thomas A Haas

**Affiliations:** 1 Department of Anatomy and Cell Biology, College of Medicine, University of Saskatchewan, Saskatoon, SK, Canada 7E3 5E5; 2 Department of Histology, Kashan University of Medical Science, Kashan, Iran

## Abstract

**Background:**

Integrins are used as prognostic indicators in breast cancer. Following engagement with extracellular matrix proteins, their signaling influences numerous cellular processes including migration, proliferation, and death. Integrin signaling varies between cell types through differential expression of integrin subunits, and changes within a given cell upon exposure to a cell agonist or through changes in its surroundings. These variations in signaling can profoundly affect the phenotypic, tumorogenecity and metastatic properties of cancer cells. In the present study, we investigated if there were differences in the expression of integrins, integrin structures, and integrin co-receptors within three breast cancer cells and if these differences effected integrin signaling.

**Methods:**

Expression of integrins, urokinase receptor and vascular endothelial cell growth factor receptor (VEGFR) in metastatic MDA-MB-435 and MDA-MB-231, non-metastatic MCF7 and non-breast cancer Hek-293 cells was measured by flow cytometry. Cell adhesion was assessed using collagen, fibrinogen, fibronectin and vitronectin coated plates. Changes in kinase levels following PMA stimulation, and cell adhesion-induced activation of kinases were determined by western blot analysis. Distribution of actin stress fibers and focal adhesions was assessed by immunocytochemistry.

**Results:**

All cells expressed α_v _integrins, while high β_5 _and α_v_β_5 _expression was restricted to the cancer cells and high β_3 _and α_v_β_3 _expression was restricted to MDA-MB-435 cells. The two metastatic cells were the least adhesive, but all cells adhered well to most proteins in the absence of PMA. All proliferating cells expressed activated pSrc, but only proliferating metastatic cells expressed high pMEK levels. PMA treatment resulted in time-dependent changes in activated kinase levels, and only MDA-MB-231 cells constitutively expressed high levels of activated pMEK. MDA-MB-435 cells formed more stress fibers and focal adhesions and only exhibited adhesion-induced activation of pMEK and pFAK. All cells expressed the urokinase receptor, but MCF7 cells had markedly higher VEGFR expression. Adhesion induced differential expression of pFAK, pMEK and pERK.

**Conclusions:**

This study demonstrates that breast cancers vary in their expression of integrins, their capacity to form focal adhesion and to signal through integrins. These differences likely contribute to phenotypic variations between cancer lines and account for some of the heterogeneity of breast cancer.

## Background

Breast cancer is one of the most common cancers and continues to rank as one of the top causes of death in women [1]. The high mortality rate associated with breast cancer is directly related to its ability to readily metastasize. Histological type, size of tumor, metastasis, epidermal growth factor receptor 2 (ErbB2) expression and lymph node involvement are key factors used to assess prognosis and probability of response to systemic therapies [2]. However, breast cancer patients undergoing treatment continue to have different clinical outcomes, despite having similar clinical diagnostic and prognostic profiles. These differences in outcomes underscore the heterogeneity of the disease, and the limitation of using a mainly morphology-based classification system for breast cancer [3]. To improve the classification of breast cancers and the use of breast cancer therapeutics, investigations into the biological mechanisms underlying breast cancer have identified new and more accurate biological markers and factors of breast cancer. Currently, cathepsin D, estrogen receptors, ErbB2, integrins, p53, urokinase plasminogen activator (uPA), uPA inhibitor-1 and urokinase receptor (uPAR) have been validated as biological prognostic markers in breast cancer [4]. Amongst these factors, integrins are a family of cell adhesion receptors that are implicated in the establishment, metastasis and progression of many cancers [5-9].

Integrins meditate cell adhesion to the cell-extracellular matrix (ECM), a fundamental cellular process that not only regulates cell growth, differentiation, and death, but also regulates malignant cell growth, metastasis and cancer-induced angiogenesis [8,10,11]. Integrins participate in these cellular processes by providing a dynamic physical linkage between the ECM and the actin cytoskeleton. Engagement of integrins with ECM ligands triggers integrin clustering, and the formation, disassembly and reorganization of actin filaments, stress fibers and focal adhesion complexes [7,12]. This dynamic reorganization of these cellular structures allows integrins to function as regulators of cell shape and cellular processes requiring cellular reshaping such as cell adhesion, cell migration and cell division. Integrin clustering and focal adhesions also elicit the activation of a number of intracellular signaling pathways to regulate cytoskeletal and ECM assembly, cell migration, proliferation, differentiation and death [7,12]. As the cytoplasmic domain of integrins lacks an actin binding domain and is devoid of enzymatic activity, all these effects are mediated by integrin associated molecules. The integrin associated adhesion proteins that participate in this integrin-actin linkage include the cytoskeletal proteins α-actinin, talin, and skelemin, and the kinases involved in integrin signaling include C-terminal Src kinase, focal adhesion kinase (FAK), integrin linked kinase, and Src [8]. FAK is a non-receptor protein tyrosine kinase that plays an important role in the localization of integrins to focal adhesions and the assembly of integrin-signaling molecules [12]. It is involved in anchorage dependent survival signaling and cell adhesion induces FAK autophosphorylation at tyrosine 397 (Y397), which creates a binding site for Src, C-terminal Src kinase, GRB7, phosphatidyl inositol 3 kinase, and phospholipase Cγ. Subsequently, Src phosphorylates FAK at a number of tyrosines including Y925 that serves as binding site for GRB2, which links integrins to the MAP kinase pathway [12]. Integrin signaling through Src can also be FAK-independent as Src also binds constitutively and directly to β_3_, and clustering of β_3 _integrins induces autophosphorylation and activation of Src [13]. The dynamics of integrin signaling is further complicated by its cross-talk with other receptors, including the breast cancer marker, uPAR, and vascular endothelial cell growth factor receptor (VEGFR) [11,14].

In this study a series of experiments were performed to better understand the role of integrin-associated proteins and structures, and integrin signaling pathways in breast cancer. A non-breast cancer line, Hek-293, and three breast cancer lines of differing metastatic and invasive capacities were used: MDA-MB-435 that are estrogen receptor-negative and highly metastatic; MDA-MB-231 that are estrogen receptor-negative and highly invasive; and, MCF7 that are estrogen receptor-positive and non-metastatic [15-17]. We determined the levels of integrins expressed by each cell line, and the capacity of a cell agonist to stimulated cell adhesion to integrin ligands and to induce intracellular signaling. We also assessed the capacity of various ECM ligands to induce heterogeneity into the formation and distribution of integrin-associated structures and proteins within the cells. Finally, we determined the levels of uPAR and VEGFR expressed by the cell lines and the capacity of cell adhesion to induce intracellular signaling via integrin-linked Src and MAPK pathways.

## Methods

### Antibodies, Reagents, Chemicals

Antibodies against β_3 _(sc-6627), Bcl2 (sc-509), c-Src (sc-8056), ERK (sc-94), FAK (sc-557), pFAK(Y397) (sc-11765), pFAK(Y861) (sc-16663), pErbB2(Y1221/Y1222) (6B12), VEGF (sc-80435), VEGFR2 (sc-57136), uPAR (sc-13522), talin (sc-7534) and HRP secondary antibodies were obtained from Santa Cruz (Santa Cruz Biotechnology, Inc., Santa Cruz, CA); β_1 _(MAB2253), β_6 _(MAB2076Z), α_v_β_3 _(LM609), α_v_β_5 _(MAB2019Z) and α_v_β_6 _(MAB2074Z) from Millipore (Millipore Canada Ltd., Etobicoke, ON); β_3 _(MHCD6100) from Invitrogen (Invitrogen Canada Inc., Burlington, ON); β_5 _(B5-IVF2) from Abcam (Abcam Inc., Cambridge, MA); MEK, pMEK (S217/S221); c-Src (36D10), pSrc(Y416) (100F9), pSrc(Y527) (2105), pMEK1/2 (9121) and pERK (197G2) from Cell Signaling (New England Biolabs Ltd., Pickering, ON); and, uPAR (MAB807) antibody from R&D (R&D Systems, Inc., Minneapolis, MN). Collagen (type I and IV), fibronectin (FN), vitronectin (VN), fibrinogen (Fg) and an antibody against vinculin (hVIN-1) were obtained from Sigma (Sigma Chemical Co., St. Louis, MO).

### Cells and Cell culture

All the cell lines were from ATCC. MDA-MB-435, MDA-MB-231, and Hek-293 cells were cultured in RMPI 1640, and MCF7 cells in F-12 containing 10% fetal calf serum and 100 U/ml penicillin and 100 μg/ml streptomycin. All cells were grown as monolayers on tissue culture plates at 37°C in a humidified incubator with 5% CO_2 _and 95% air. Cells were subcultured at 80-95% confluence using 0.25% trypsin (w/v)/5 mM EDTA to detach cells.

### Flow cytometry

Cells were grown in 100 mm tissue culture plates to 90-95% confluence and harvested with 2% EGTA. For measurement of integrin expression, once harvested all samples were maintained at 4°C to maintain the expression of integrins on the cell surface. Thus, cells were washed and re-suspended in 4°C Tyrode-Hepes Buffer containing 1 mM CaCl_2_, 1 mM MgCl_2_, 5.5 mM Glucose and 1 mg/ml BSA. Cells were incubated with primary antibodies for one hour at 4°C, washed three times with ice-cold Tyrode-Hepes Buffer and incubated with PE or Alexa Fluor-488 labeled secondary antibody for another one hour at 4°C. Cells were washed, re-suspended in 0.5 ml of ice-cold Tyrode-Hepes Buffer and kept on ice until analyzed by flow cytometry. Isotype-matched monoclonal antibodies were used as controls. For phorbol 12-myristate 13-acetate (PMA) treatment, cells were grown for 16 hours in media containing 1% fetal calf serum and then the cells were treated with 150 nM PMA for two hours. For mock treatment, the cells were incubated with the same concentration of DMSO as was present in the PMA samples. Data was analyzed using Flowjo program.

### Adhesion Assay

Adhesion assays were performed as previously described with minor modifications [18,19]. Briefly, 96-well plates were coated with 20 μg/ml of collagen, FN, Fg or VN overnight at 4°C. The wells were blocked with 2% BSA and washed with PBS. MDA-MB-435, MDA-MB-231, MCF7 or Hek-293 cells were suspended in serum free media, with or without the addition of 150 nm PMA. The cells were then transferred to the wells (2 × 10^5 ^cells/well) and incubated for one hour at 37°C. Unattached cells were removed by washing with PBS and the cells were then incubated in staining solution (20% methanol, 1% formaldehyde and 0.5% crystal violet in H_2_O) for 30 min. Plates were washed, lyzed in 0.5% Triton X-100, and adhered cells quantitated by measuring light absorbance at 590 nm.

### Western blotting

Cells were grown to 90-95% confluence, washed with ice-cold PBS and lyzed in 500 μl of RIPA buffer (50 mM Tris, pH 8, 150 mM NaCl, 0.1% SDS, 0.5% Na deoxycholic Acid, 1% NP-40 or IGEPAL, 10 μg/ml aprotinin and 10 μg/ml leupeptin), and using a 25 gauge needle. Cell extracts were centrifuged and supernatants kept at -20°C. Equal amounts of protein (24 μg/well) were electrophoretically separated in SDS polyacrylamide gels and proteins were transferred to a nitrocellulose membrane. Membranes were blocked with 5% skim milk and probed with primary antibodies, followed by incubation with HRP-labeled secondary antibodies. Western blots were visualized by an enhanced chemiluminescence detection system according to the manufacturer's protocol (Amersham Life Sciences, Arlington Heights, IL).

### Immunofluorescence

Falcon 4-well CultureSlides were treated with 1% SDS, rinsed with PBS and then coated overnight at 4°C with 20 μg/ml of collagen, FN, Fg or VN. Cells were seeded and grown overnight on different ligand-coated chamber cells. Cells were fixed with 4% paraformaldehyde for 10 min, permeabilized with 0.2% (v/v) Triton X-100, washed and then blocked with 1% BSA. Filamentous actin (F-actin) was stained using Alexa Fluor 594 phalloidin (Invitrogen, San Diego, CA) for 30 min at a dilution of 1:40. Focal adhesions were stained using an antibody to vinculin (Sigma Chemical Co., St. Louis, MO), or to talin (Santa Cruz Biotechnology, Inc., Santa Cruz, CA) at a dilution of 1:100 and a fluorescein-conjugated secondary antibody.

## Results

### Integrin expression

**Figure 1:**
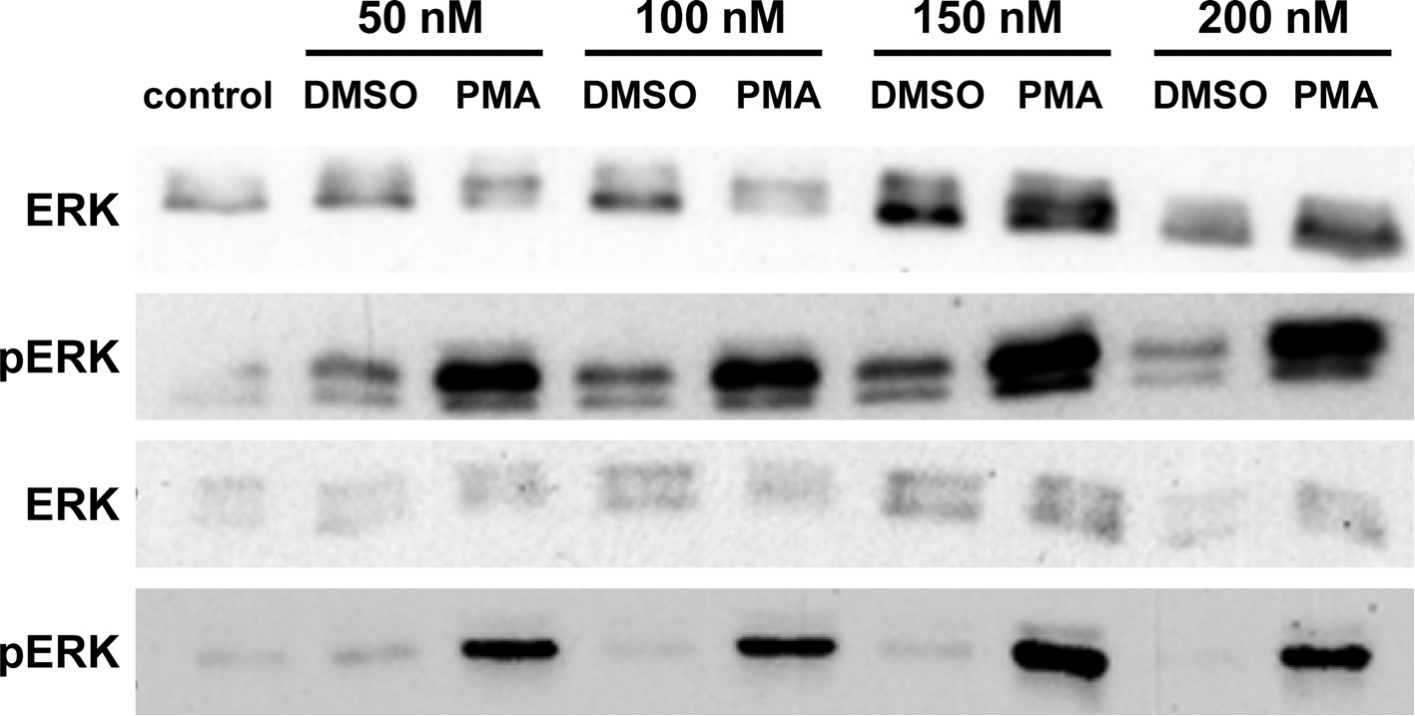
**Optimal PMA treatment concentration for pMEK and pERK activation**. Cells were plated and grown overnight in media containing 1% fetal calf serum, except for control cells that were grown in regular media containing 10% fetal calf serum. Cells were then treated with increasing amounts of PMA (50-200 nM) for two hours. Additional control cells were incubated for one hour with the same concentration of DMSO as present in the PMA samples (DMSO). Cells were lyzed, and equal amounts of total protein of each sample were subjected to immunoblotting with antibodies against ERK and pERK. Two representative experiments are shown.

**Figure 2:**
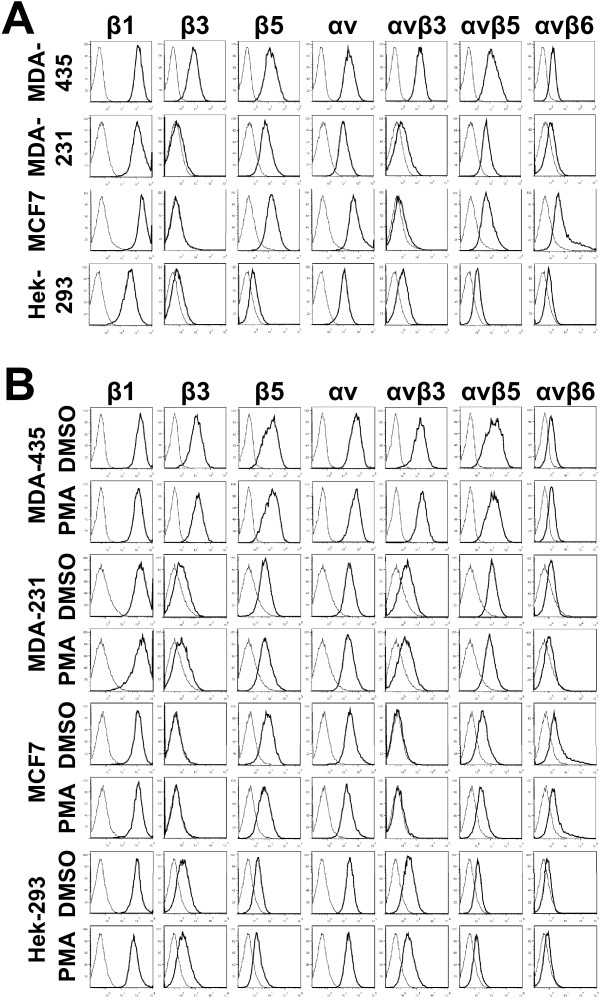
**Integrin expression by breast cancer and Hek-293 cells**. A) Flow cytometric analysis of untreated cultured cells. Cells adhered to FN-coated dishes were harvested and incubated with the mouse anti-human β_1_, β_3_, β_5_, α_v _,α_v_β_3 _,α_v_β_5 _and α_v_β_6 _antibodies, followed by incubation with fluorescent-labeled secondary antibody and then analyzed by flow cytometry (*black lines*). Isotype-matched irrelevant antibodies were used as controls (grey *lines*). B) Flow cytometric analysis of integrin expression by PMA stimulated cells. Adhered cells were incubated overnight in media containing 1% fetal calf serum and then stimulated with 150 nM PMA for two hours. The cells were then harvested and integrin expression levels assessed as described in panel A. In control plates (mock treatment), an equal quantity of DMSO was added instead of PMA. One of three representative experiments is shown.

Previous studies have identified a linkage between the expression of β_1 _and α_v _integrins and breast cancer [4,6]. In addition, cell agonists such as PMA that activate protein kinase C and induces phosphorylation of pERK, promote integrin-mediated cell adhesion, focal adhesion formation and cell signaling in many cell types including cancer cells [19,20]. Therefore, we first identified an optimal concentration of PMA that induced pERK formation (Figure 1) and then assessed the relative levels of these integrins expressed by adhered breast cancer cells and Hek-293 cells using flow cytometry of untreated (Figure 2A) and PMA treated cells (Figure 2B). To determine the optimal concentration of PMA to use, MDA-MB-435 cells were stimulated with different concentrations of PMA (50 to 200 nM) and then the level of pERK was determined by western blot analysis (Figure 1). Results indicated that 150 nM PMA produced the highest levels of pERK, in agreement with our previous studies using similar concentrations of PMA as an activator of cell adhesion in other cell lines [18,19]. Therefore, 150 nM PMA was used as the PMA stimulus in the remaining experiments.

To maintain the integrity of the surface expression of integrins on cell adhered to FN, all cells washes and incubations were performed at 4°C prior to their analysis by flow cytometry. We consistently found that the non-breast cancer cell line, Hek-293, generally expressed lower integrin levels as compared to the three breast cancer lines (Figure 2A). Hek-293 expressed very low levels of β_3_, β_5_, α_v_β_3_, α_v_β_5 _and α_v_β_6_, but higher levels of β_1 _and α_v_. All three breast cancer cell lines expressed high levels of β_1 _and α_v_, and they also expressed higher levels of β_5 _and α_v_β_5 _in comparison to Hek-293. MDA-MB-435 integrin expression distinguished this cell line from all others as they consistently expressed higher levels of integrins and they were the only cell line to express high levels of β_3 _and α_v_β_3_.

Next, the effect of short-term PMA stimulation on integrin expression in the cancer and Hek-293 cells was evaluated (Figure 2B). The results obtained for PMA treated cells were nearly identical to those of mock DMSO treated cells and untreated cells (panel A). Integrin expression remained unchanged or was only slightly altered by PMA treatment. These results are consistent with previous findings that short-term PMA treatment does not enhance integrin expression [20], rather it activates integrins [18]. In addition, we determined that short-term suspension or adhesion of cells in the presence or absence of PMA did not affect integrin expression (experiments performed in duplicate or triplicate). For example, expression of α_v_β_3 _in MDA-MB-231 suspension cells treated with DMSO or PMA was 9.7% and 9.9%, respectively, and expression of α_v_β_3 _in two hour adhered MDA-MB-231 cells was 2.5% and 2.8%. Furthermore, the expression of α_v_β_3 _in MDA-MB-435 suspension cells treated with DMSO or PMA was 99.1% and 98.2%, respectively, and expression of α_v_β_3 _in two hour adhered MDA-MB-435 cells was 98.4% and 98.8%.

### Adhesion of breast cancer cell lines

**Figure 3:**
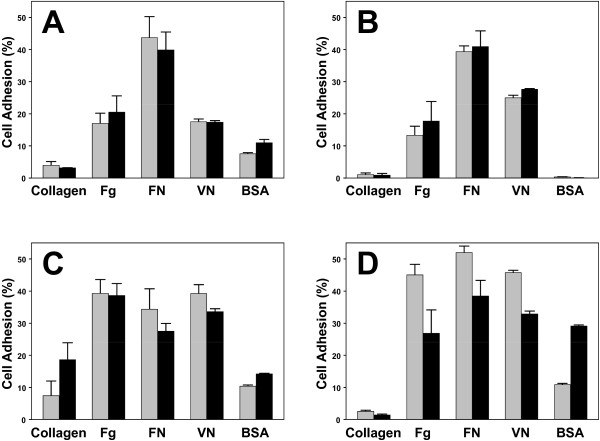
**Cell adhesion to various ECM integrin ligands**. Cells (A, MDA-MB-435; B, MDA-MB-231; C, MCF7; and D, Hek-293) were harvested and then untreated (grey box) or 150 nM PMA-treated (black box) cells were allowed to adhere to the plates coated with various ECM proteins for 1 hour. The plates were washed and the number of adhered cells was calculated as a percentage of total added. Mock treated cells, incubated with the same concentration of DMSO as was present in the PMA samples, had similar levels of adhesion as untreated cells (data not shown). One of four representative experiments performed triplicate is shown.

Cell adhesion plays a vital in the survivability and progression of a cancer as engagement of integrins with the ECM prevents some cancers from undergoing apoptosis while it induces cell proliferation in others. In metastatic cancers, cell adhesion undergoes rapid regulatory changes that allow the cancer cell to disengage from the ECM, migrate and then reengage with the ECM at its secondary metastatic site. In addition, short-term exposure of cells to cell agonists such as PMA, results in increased α_v _integrin-mediated cell adhesion and spreading onto ECM proteins [19,20]. Therefore, we assessed the capacity of 150 nM PMA to influence the adherence of the breast cancer cells to ECM proteins (Figure 3). We used FN, Fg and VN as ligands with differing specificity for α_v _integrins and collagen as a non-α_v _integrin ligand. In general, the adhesion of unstimulated cells, cells incubated in media alone, was markedly greater than we previously reported for GM1500 or M21 cancer cells [18,19], with 20 to 40% of the total cells adhering within one hour. The majority of cells that adhered within one hour were firmly attached and cell spreading (formation of lamellipodia and filopodia) was readily detected (data not shown). Unstimulated MDA-MB-435 (Figure 3A) and MDA-MB-231 (Figure 3B) cell adhered highest to FN, while MCF7 (Figure 3C) and Hek-293 cells (Figure 3D) had equal preference for FN, Fg and VN. MDA-MB-231 showed the lowest nonspecific binding to BSA, and MCF7 cells were the only cell line that adhered well to collagen. However, in contrast to our previous studies using α_v_β_3_-expressing GM1500 cancer cells [18], PMA treatment did not upregulate cell adhesion. Increasing the PMA treatment and adhesion time to four hours also showed no PMA effect (data not shown). The adhesion of mock treated cells, incubated with the same concentration of DMSO as was present in the PMA samples, were also similar to that of unstimulated cells (data not shown). Therefore, we tested the hypothesis that the non-PMA treated cells were already near maximal levels of adhesion which negated any further increase with PMA treatment. Using GM1500 cells, we observed that less than 5% of the non-treated cells adhered to Fg, and the cell adhesion increased two to four-fold following PMA treatment (data not shown). These results led us to conclude that the breast cancer and Hek-293 cells expressed an integrin co-receptor or a non-integrin adhesion receptor that upregulated or directly facilitated cell adhesion. To determine to what extent the adhesion was mediated by integrins, the cells were allowed to adhere to FN for one and two hours in the absence and presence of α_v _and β_1 _functional-blocking antibodies. The adhesion of MDA-MB-435, MDA-MB-231, MCF7 and Hek-293 cell after one hour was inhibited 79.1% ± 8.8; 79.8% ± 8.4; 42.3% ± 24.5; 80.7% ± 8.7 (mean ± stdev), respectively by the addition of both antibodies (n = 5 in duplicate experiments). At two hours the adhesion was inhibited 82.5% ± 7.25; 75.4% ± 11.4; 64.5% ± 14.7; and, 90.2% ± 4.9, respectively. Thus, MDA-MB-435, MDA-MB-231 and Hek-293 cell adhesion was highly integrin-mediated, while only two-thirds of MCF7 adhesion was integrin-mediated. This led us to speculate that the increase in adhesive capacity of these cell lines was a result of increased integrin activation through the action of either a co-receptor or upregulated signaling through intracellular pathways.

### Agonist-induced signaling

**Figure 4:**
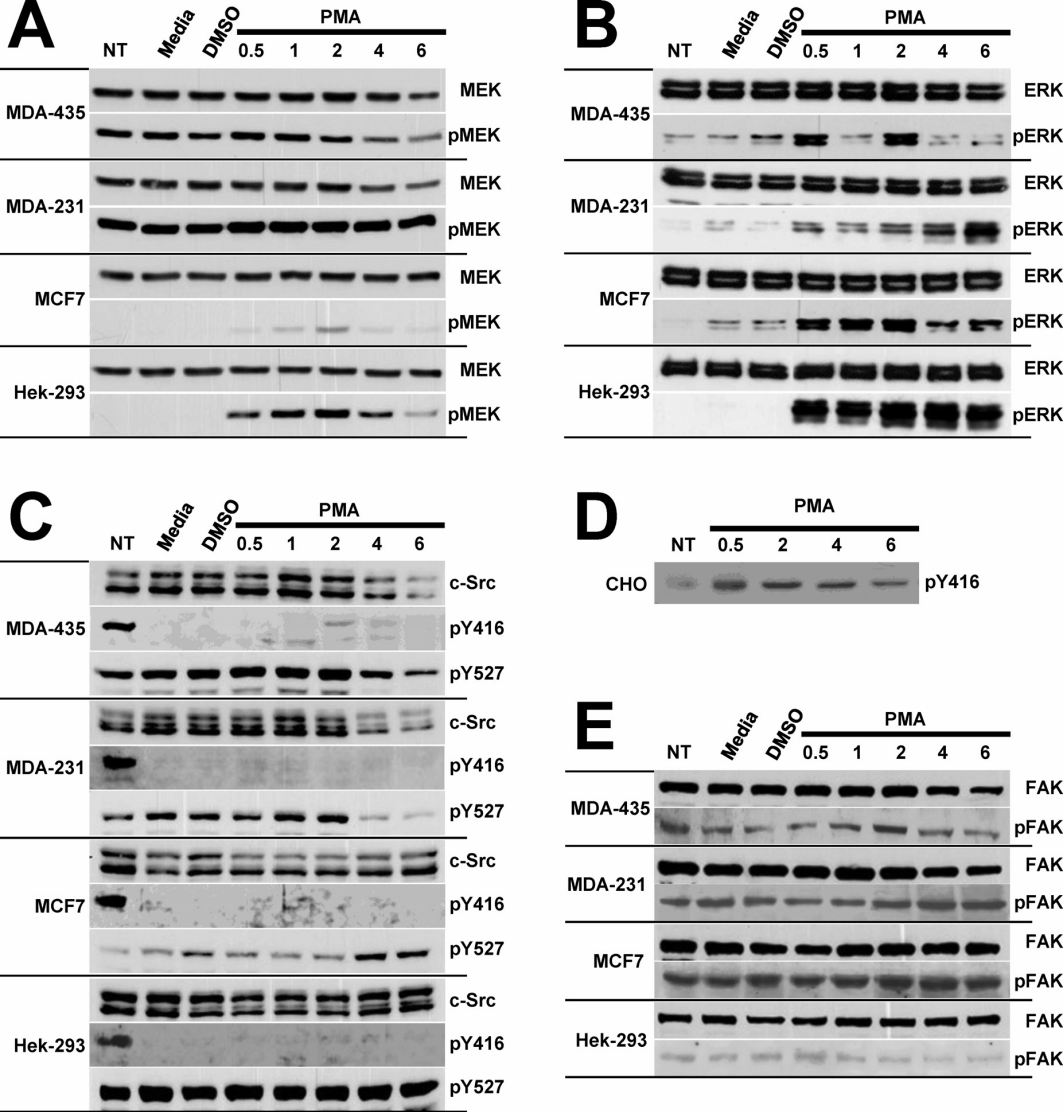
**Effect of PMA treatment on the expression of signaling proteins**. All cells were plated and grown overnight in media containing 1% fetal calf serum, except for a control cells that received no treatment (NT) and were grown in media containing 10% fetal calf serum. Cells were then treated with 150 nM PMA for times indicated. Additional control cells were incubated for one hour with either media alone (Media), or with the same concentration of DMSO as was present in the PMA samples (DMSO). Cells were lyzed, and equal amounts of total protein of each sample were subjected to immunoblotting with antibodies against (A) MEK and pMEK; (B) ERK and pERK; (C) c-Src, pSrc(Y416) (pY416) and pSrc(Y527) (Y527); or (E) FAK and pFAK. Western blots of (D) pSrc(Y416) in Chinese hamster ovary (CHO) cells expressing α_IIb_β_3 _and adhered to Fg are also displayed for comparison. One of three representative experiments is shown.

Cells continuously respond to their extracellular environment and cues provided by ECM proteins, growth factors, cytokines and other cell agonists can invoke varying responses within different cell types. Thus, some of the heterogeneity of breast cancer could be a result of varying responses by different breast cancer cells. Therefore, we determined if all the breast cancer cells responded in a similar manner to a cell agonist. Furthermore, as integrins are responsible for transmitting signals from the environment to the cell, we also determined if the high adhesion of unstimulated breast cancer cells resulted in upregulated intracellular signaling. We therefore allowed the cells to adhere overnight onto FN-coated plates and then measured the levels of integrin signaling molecules before and for various times after treatment with 150 nM PMA. MEK levels were unchanged by PMA treatment in MCF7 and Hek-293 cells, and only decreased in MDA-MB-435 and MDA-MB-231 cells after two hours of treatment (Figure 4A). However, marked changes occurred in the levels of activated pMEK (S217/S221). In MDA-MB-435 cells, pMEK levels in untreated and PMA treated cells remained high until 2 hours of PMA treatment and then decreased, while in MDA-MB-231 cells pMEK levels remained higher and unaltered by PMA treatment. The pattern of pMEK expression in MCF7 cells was markedly different from the metastatic cells. All non-PMA treated MCF7 cells (lanes 1-3) containing undetectable levels of pMEK, and only a weak transient signal was detected following PMA treatment. The pattern of pMEK expression in Hek-293 was similar to that of MCF7 cells. Furthermore, regardless of the differences in pMEK levels following PMA treatment, high pMEK levels in adhered MDA435 and MDA231 cells separated these metastatic cells from the non-metastatic MCF7 and Hek293 cells.

PMA treatment had no effect on the high levels of ERK present in each cell line (Figure 4B). In contrast, the levels of activated pERK were very low in most of the non-treated cells and PMA treatment resulted in differential upregulation of pERK. The levels of pERK in MDA-MB-435 cells transiently increased in a biphasic response to PMA, reaching maxima at 30 min and two hours. In MDA-MB-231 cells, pERK levels never reached a maximum, while pERK levels in MCF7 cells increased between 30 min and two hours. There was high and sustained induction of activated pERK in Hek-293 cells following PMA treatment (Figure 4B). Thus, there was heterogeneity in MAPK pathway signaling by adhered breast cancer cells in the absence and presence of PMA.

The Src pathway was investigated in the cells by evaluating their levels of c-Src, activated Src [pSrc(Y416)] and deactivated Src [pSrc(Y527)]. The levels of c-Src remained unchanged in MCF7 and Hek-293 cells, while they decreased after two hours of PMA treatment in the metastatic MDA-MB-435 and MDA-MB-231 cells (Figure 4C). PMA induced activation of Src in MDA-MB-435 cells, with pSrc(Y416) levels reaching at maxima at two hours. There was minimal induction of pSrc(Y416) in MDA-MB-231, MCF7 and Hek-293 cells. In addition, all cells grown in media containing 10% fetal calf serum that supports cell proliferation (lane one) contained higher levels of activated pSrc(Y416) than when grown in 1% fetal calf serum (lanes 2-7). This cell proliferation effect was not observed for any of the other signaling proteins examined. To confirm that these cell lines expressed low levels of activated pSrc in 1% fetal calf serum, we also measured the level of pSrc(Y416) in α_IIb_β_3_-expressing Chinese hamster ovary (CHO) cells adhered to Fg (Figure 4D). Here, pSrc(Y416) levels were readily detected and upregulated. The levels of deactivated pSrc(Y527) in MDA-MB-435 and MDA-MB-231 cells also reached a maximum at two hours, while they increased in MCF7 cells after two hours. In contrast to the cancer cells, Hek-293 cells expressed high and unaltered levels of deactivated Src.

FAK levels remained unchanged in all cell lines, except after two hours of treatment in MDA-MB-435 cells (Figure 4E). The levels of activated pFAK also remained unaltered in MCF7 and Hek-293 cells but did transiently increase at two hours in MDA-MB-435 cells, which correlated with maximal levels of pERK and pSrc(Y416). MDA-MB-231 pFAK levels increased after one hour which correlated only with their pERK levels. Therefore, we observed heterogeneity in MAPK and Src signaling by the breast cancer cells.

### Immunocytochemistry

**Figure 5:**
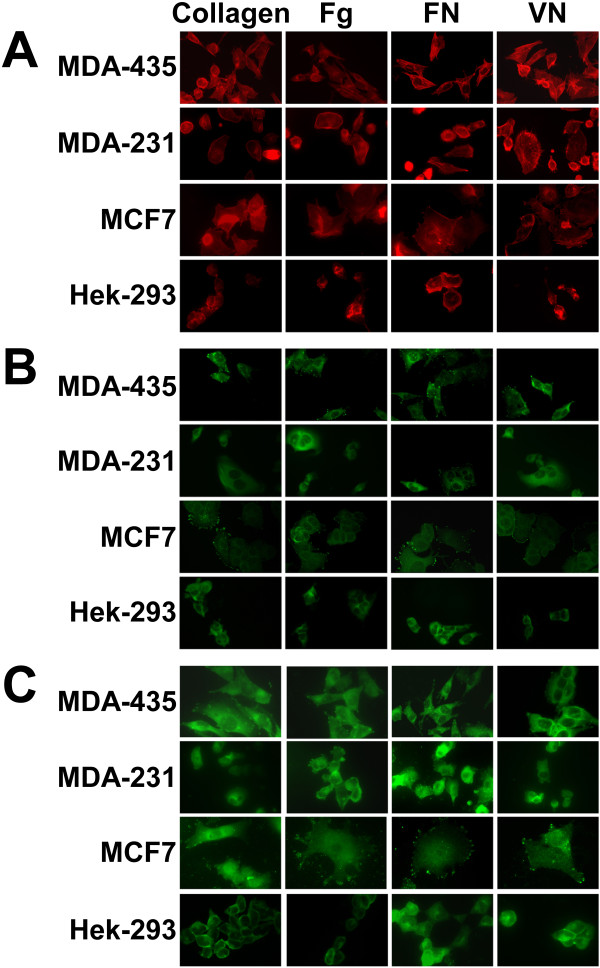
**Immunocytochemistry of actin stress fibers and focal adhesions**. Chamberslides were coated with 20 μg/ml of collagen, Fg, FN or VN and then MDA-MB-435, MDA-MB-231, MCF7 or Hek-293 cells (1 × 10^5^/slide) were seeded and allowed to adhere overnight. The slides were washed, fixed, permeabilized and then stained for F-actin (A), vinculin (B) and talin (C). One of four representative experiments is shown.

Integrin signaling is complex as it not only governed by the binding of an ECM ligand but it is also regulated by the recruitment and interaction of integrin-associated proteins with integrin clusters and the formation of integrin-based structures, such as focal adhesions. As adhered breast cancer cells differed in their signaling (Figure 4), we investigated if these differences in signaling were due to changes in integrin-based structures. Therefore, experiments were performed to determine whether the differences were due to changes in the subcellular distribution of F-actin stress fibers or the formation of focal adhesions when the cells were allowed to attach to and spread on ECM ligands (Figure 5). The cells were plated onto coverslips coated with collagen, Fg, FN or VN, and allowed to adhere overnight. Cells were fixed, permeabilized, and stained for F-actin and focal adhesions. F-actin stress fibers were easy to identify and major differences in the distribution and organization of F-actin fibers were observed (Figure 5A). In MDA-MB-435 cells adhered to the four ECM ligands, many bundles of stress fibers spanning the core of the cells were observed, and adherence to FN and VN induced the greatest formation of stress fibers. In MDA-MB-231 cells, F-actin was mainly present at the perimeter of the cell and localized to membrane protrusions resembling filopodia. When grown on FN and VN, MDA-MB-231 cells contained more and denser clustering of the protrusions than MDA-MB-435 cells. The distribution of F-actin in MCF7 was condensed and localized to the leading edge of spreading cells. In contrast, Hek-293 cells were almost devoid of stress fibers.

Vinculin is a prominent component of focal adhesions and it induces integrin clustering and focal adhesion formation through interactions with talin, an actin-integrin linkage protein [21]. Therefore, focal adhesions were visualized using vinculin staining (Figure 5B). Compared to the three other cell lines, MDA-MB-435 adhered to the four ECM ligands show enhanced focal adhesion formation, which correlated with the presence of strong stress fibers. Some focal adhesions were found distributed at the periphery of MCF7 cells, while only FN induced the formation of a few focal adhesions in MDA-MB-231 cells. No focal adhesions were detected in Hek-293 cells.

The staining pattern with anti-talin was similar to that of vinculin (Figure 5C). As talin is reported to be both an integrin-linkage protein and an integrin activator [22], its recruitment to focal adhesions also serves as a mechanism for focal integrin activation and signaling. In MDA-MB-435 and MCF7 cells adhered to any of the ligands, talin staining revealed a diffuse distribution of talin within the cytoplasm and a strong recruitment of talin to focal adhesions localized to lamellipodia and filopodia. In MDA-MB-231 cells adhered to collagen, Fg and VN, very few focal adhesions were detected using talin staining. However, a dot-like distribution pattern resembling focal complexes was observed in MDA-MB-231 cells adhered to FN. Hek-293 cells did not form any focal adhesions and cell spreading was much higher on FN than on the other ligands. Therefore we observed that MDA-MB-435 cells expressed the highest level and organization of actin-integrin linkage structures and focal adhesions. The higher level of focal adhesions in the MDA-MB-435 cells is consistent with our observation that this cell line had the strongest correlation between PMA-induced activation of pFAK, pSrc(416) and pERK (Figure 4). Furthermore, our MDA-MB-435 data is consistent with previous findings that higher expression levels of integrin αvβ3, are associated with well-developed focal adhesions and thicker stress fibers in primary breast cancer cells compared with the normal breast epithelial cells [23]. Finally, we also observed that a two hour treatment of cells with PMA induced stress fiber perturbations in all cell lines, loss of focal adhesions in MDA-MB-435 cells and induced some MCF7 cells into apoptosis (data not shown).

### uPAR and VEGFR expression

**Figure 6:**
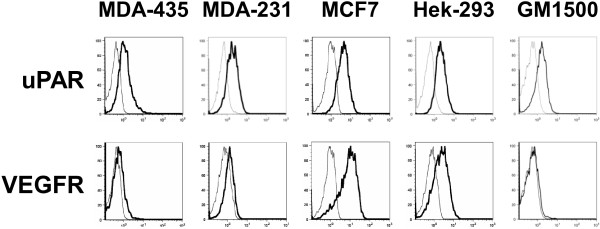
**uPAR and VEGFR expression**. The level of uPAR and VEGFR expression (black lines) in unstimulated MDA-MB-435, MDA-MB-231, MCF7, Hek-293 cells and GM1500 cells were assessed by flow cytometry using anti-uPAR and VEGFR antibodies as described in Figure 1. Isotype-matched irrelevant antibodies were used as controls (grey *lines*). One of three representative experiments is shown.

Integrin signaling is a dynamic process, being influenced by a number of factors including the cross-talk with other cell surface receptors, such as uPAR and VEGFR. These two receptors are also implicated in breast cancer tumor progression and invasiveness. Signaling by uPAR requires interactions with integrin or other co-receptor as it lacks a transmembrane and an intracellular domain [14]. uPAR also contributes to breast cancer development by directly supporting cell adhesion to VN, and by coordinating ECM proteolysis and remodeling through activation of plasmin and breakage of integrin-ECM linkages that allow for cell migration and metastasis [14]. The interaction of VEGFR with integrins, such as α_v_β_3_, α_v_β_5 _and α_5_β_1_, is involved in cancer-induced angiogenesis that facilitates the growth and progression of breast cancers [11]. Therefore, the levels of uPAR and VEGFR expressed by the cell lines were determined (Figure 6).

The breast cancer and Hek-293 cells all expressed uPAR, with MCF7 expressing slightly higher levels of uPAR than MDA-MB-231 and MDA-MB-435 cells (Figure 6). As all cells, and in particular MCF7 cells, adhered well in the absence of an agonist (Figure 3), we questioned whether uPAR may have been involved in the upregulated adhesion. To address this question we also determined the levels of uPAR in GM1500 cells which we demonstrated had low adherence in the absence of a cell agonist. However, we found that uPAR levels in GM1500 cells were similar to those of MDA-MB-231 and Hek-293 cells (Figure 6). This led us to conclude that the levels of uPAR expressed in MDA-MB-231 and Hek-293 cells were insufficient to upregulate cell adherence. In contrast to uPAR expression, VEGFR expression varied greatly between the cell lines (Figure 6). MCF7 cells expressed greater than 10-fold more VEGFR compared to MDA-MB-435 and GM1500 cells, while MDA-MB-231 and Hek-293 cells expressed low to moderate amounts, respectively. In addition, we determined that all cell lines produced very low amounts of VEGF (data not shown). Thus, MCF7 cells were readily distinguished from the metastatic cells based upon their expression of VEGFR.

### Adhesion-induced differential signaling

**Figure 7:**
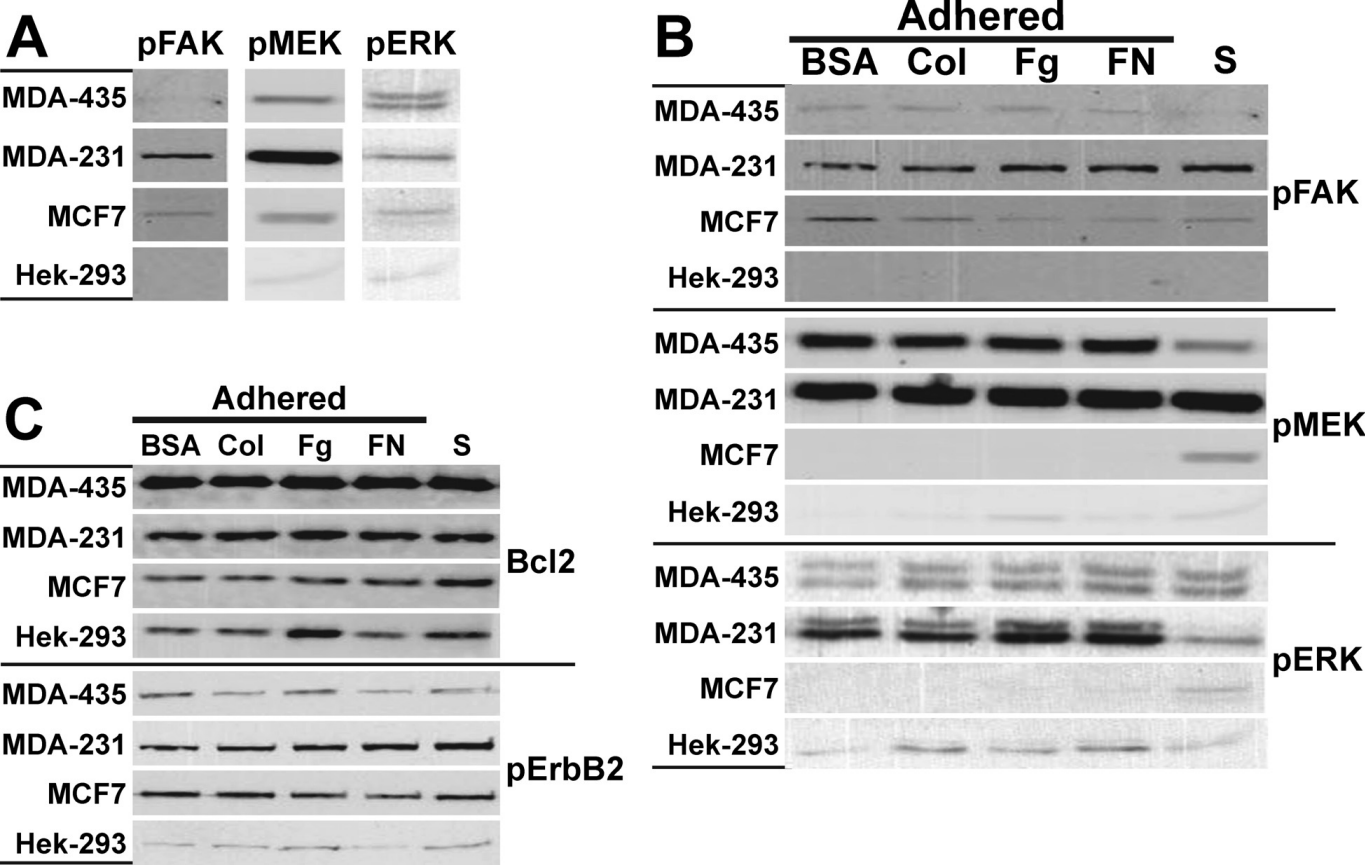
**Adhesion induced signaling of Hek-293 and breast cancer cells**. Cells were either left in suspension or placed into wells coated with BSA, collagen (Coll), Fg and FN. After a one hour incubation at 37°C, plates were washed to remove non-adhered cells. Total cellular protein was then extracted from adhered and suspension cells and subjected to immunoblotting with antibodies against pFAK, pMEK and pERK as described in Figure 3. Blots for suspension cells are displayed in panel A. Blots for suspension and adhered cells are displayed in panel B. FAK, MEK, ERK, c-Src and pSrc levels were also assessed and remained unchanged (data not shown). (C) Samples were also probed using antibodies against Bcl2 and activated pErbb2. One of three representative experiments is shown.

During the adherence of a cell to the ECM, integrins interact with a number of matrix and cellular proteins that result in the activation of signaling pathways resulting in changes in cellular function and biology. As the breast cancer cells used in this study differed in their capacity to form focal adhesions, we explored the possibility that part of the heterogeneity of breast cancer was due to variations in adhesion-induced signaling through MAPK and Src pathways by different breast cancers. In looking at the Src pathway, we discovered that Src was highly deactivated in all cell lines and that the level of pSrc(Y527) and c-Src were unchanged by adherence to ECM proteins (data not shown). Therefore, we focused our attention on the MAPK pathway by first ascertaining if there was constitutive signaling from integrins through to ERK by measuring the levels of pFAK, pMEK, and pERK in non-adherent suspension cells (Figure 7A). All cancer cells contained activated pFAK, pMEK, and pERK in suspension, with MDA-MB-231 cells expressing much greater levels of pFAK and pMEK. Hek-293 suspension cells contained very low levels of pMEK and pERK, and typical of a nonadherent cell, they contained undetectable levels of pFAK.

As MDA-MB-231 suspension cells expressed the highest levels of pFAK and pMEK, but MDA-MB-435 expressed the highest levels pERK, we further investigated the differences in their regulation of MAPK pathway using adhered cells (Figure 7B). Adhered MDA-MB-231 cells contained higher levels of pFAK compared to MDA-MB-435 cells, but only MDA-MB-435 cells exhibited a slight but reproducible adhesion-dependent increase in pFAK. This result was consistent with MDA-MB-435 cells containing more focal adhesions than MDA-MB-231 cells (Figure 5). Adhesion of MCF7 cells to ECM ligands resulted in only small changes in pFAK, while Hek-293 cells contained no pFAK (Figure 7B). The absence of activated pFAK in Hek-293 cells was consistent with this cell line containing no focal adhesions. The levels of pMEK and pERK in non-metastatic MCF7 cells clearly distinguished this cell line from the metastatic MDA-MB-435 and MDA-MB-231 cells. Adhered MCF7 cells contained nearly undetectable levels of pMEK and pERK, while MDA-MB-435 and MDA-MB-231 cells contained high levels of both these proteins. Most adhered Hek-293 cells contained low but detectable levels of pMEK and pERK, and pERK levels increased following adhesion.

Adhesion-induced changes in pMEK and pERK levels also distinguished MDA-MB-435 from MDA-MB-231 cells (Figure 7B). There was an adhesion-dependent increase in pMEK levels in MDA-MB-435 cells, but not in MDA-MB-231 cells. It also appeared that there was constitutive activation of pMEK in MDA-MB-231 cells, as the level of pMEK in suspension cells were similar to those found in adhered MDA-MB-231 and MDA-MB-435 cells. However, once again, high pMEK levels in adhered metastatic MDA435 and MDA231 cells separated these cells from non-metastatic MCF7 and Hek293 cells. The effects of adhesion on the level of pERK in MDA-MB-435 and MDA-MB-231 cells contrasted those of pMEK. Here we observed an adhesion-dependent increase in pERK levels in MDA-MB-231 cells, but not in MDA-MB-435 cells. These differences were not due to changes in total FAK, MEK or ERK levels which remained unaltered (data not shown). As ERK is immediately downstream from MEK, we speculate that the differences in pERK levels were due to differences in the regulation of pERK-related phosphatase activity within these cells. In MDA-MB-231 cells, we propose that adhesion suppresses phosphatase activity allowing for pERK levels to increase, while in MDA-MB-435 cells, either adhesion increases phosphatase activity or pERK levels in suspension cells are already at maximal. Whatever explanation is correct, there were differences in MAPK signaling between MDA-MB-435 and MDA-MB-231 cells and a marked reduction in MAPK signaling by MCF7 cells. We also noted that there are likely other non-integrin receptors involved in cell adhesion-induced signaling as adhesion to BSA resulted in increased pFAK, pMEK and pERK levels in some cell lines.

We also examined the effect of cell adhesion on Bcl2 and pErb2 levels. Bcl2 is an important regulator of apoptosis and Bcl2 itself is regulated by integrin signaling. pErbB2 is involved in signal pathways leading to cell growth and differentiation which are two cellular processes regulated by integrin signaling. Therefore, we determined the effect of cell adhesion on Bcl2 and pErb2 levels to identify any correlations in changes in their levels to that of pMEK, pERK or pFAK. Bcl2 levels were unaffected by cell adhesion, and similar to the levels of phosphorylated kinases, no major differences in Bcl2 levels were found in cells adhered to FN versus Fg or collagen. MDA-MB-435 expressed the highest levels Bcl2, but expressed the lowest level of activated pErbB2. MDA-MB-231 and MCF7 cells expressed similar amounts of pErbB2 while Hek-293 cells expressed the lowest, in agreement with pErbB2 being a prognostic marker for some breast cancers.

## Discussion

Integrins play an important role in cell anchorage, migration, differentiation and death [5,7], and their upregulated expression in human cancers frequently indicates poor prognosis. Although breast cancer is a heterogeneous form of cancer, α_v _integrins as well as other proteins have been identified as prognostic markers. In the present study, using two metastatic (MDA-MB-435 and MDA-MB-231) and a non-metastatic (MCF7) breast cancer cell line, we demonstrated that α_v _integrin expression varies between the cell lines (Figure 2A). This variation may partially account for the heterogeneity that is found in breast cancer. In comparison to the non-breast cancer Hek-293 cells, all the cancer cells expressed higher but varying levels of β_5_, α_v_β_5 _and α_v_β_6_. Normal epidermal cells express α_v_β_5 _but after transforming into squamous carcinomas, the expression of α_v_β_5 _is down-regulated and α_v_β_6 _up-regulated that protects the cancer from undergoing anoikis [24]. Thus, differences in α_v_β_5 _and α_v_β_6 _expressions may account for some of the heterogeneity in the phenotypes of breast cancers. Furthermore, we found that only MDA-MB-435 cells expressed high levels of β_3 _and α_v_β_3_. In vivo studies reveal that αvβ3 is also involved in enhanced metastasis of breast cancer to bone [25]. The high levels of β_3 _and α_v_β_3 _in metastatic MDA-MB-435 cells is in keeping with β_3 _being an important mediator of melanoma cell invasion and migration and with α_v_β_3 _as a prognostic indicator in breast cancer [4,5,26,27]. However, as MDA-MB-231 and MCF7 cells did not express α_v_β_3_, α_v_β_3 _should not be viewed as a universal prognostic indicator for all forms of breast cancer. Rather, it should be used as an indicator where the use of anti-α_v_β_3 _therapeutics is warranted.

Integrins, play a significant role in the acquisition and maintenance of neoplastic phenotype by preventing apoptosis and maintaining cell proliferation, and integrin expression profile can dramatically change upon the normal-to-neoplastic transition [6]. However, we found that short term (one to two hours) of adhesion onto FN or Fg had minimal effect on integrin expression in MDA-MB-432, MDA-MB-231 and MCF7 cells. Thus, it is likely that changes in integrin expression profile during cancer cell metastasis may either require more time or may also require the activity of matrix-degrading proteases, such as uPA and matrix metalloprotease 2, to modify the surrounding tissue [5].

In nonmalignant and cancer cells, integrin-mediated adhesion of unstimulated cells is usually low and can be upregulated by the addition of a cell agonist, such as PMA [18,19]. In this study, we found that the adhesion of unstimulated breast cancer and Hek-293 cells was already upregulated, and that level of uPAR expressed by the cells (Figure 6) was likely not sufficient enough to upregulate cell adhesion. However, all cell lines when adhered and proliferating constitutively expressed activated pSrc (Figure 4C, lane 1), which may have been influenced by uPAR-integrin interaction, or in MDA-MB-435 and Hek-293 cells, partially a result of Src signaling following its direct binding to β_3 _[13,14]. Adhesion to VN is mediated by uPAR [14] and by a number of integrins including α_v_β_1_, α_IIb_β_3_, α_v_β_3_, α_v_β_5_, α_v_β_6 _and α_v_β_8 _[28]. Similarly, other integrins also share common ligands, which likely accounts for why we did not observe a strong preference for one ECM ligand. In addition, non-integrin adhesion receptors also contributed to cell anchorage as all cells, except MDA-MB-231, adhered to BSA.

The formation of focal complexes, focal adhesion and other integrin-related cellular structures has a profound effect on cell shape and numerous cellular processes that govern the biology of a cell [12]. Our vinculin and talin staining produced similar results which agree with the role of vinculin in controlling focal adhesion formation by directly interacting with talin [21]. F-actin and focal adhesion staining demonstrated that the non-breast cancer cell line, Hek-293, was nearly devoid of integrin-associated structures in comparison to the breast cancer lines (Figure 5). We also observed that a two hour PMA treatment induced stress fiber perturbations in all cell lines, and resulted in a loss of focal adhesions in MDA-MB-435 cells. These results are consistent with previous findings that PMA-mediated F-actin reorganization and redistribution is closely linked with cell transformation [29]. We also concluded that some of the heterogeneity of breast cancer can be explained by variations in the level of integrin-associated F-actin structures between different breast cancers. MDA-MB-435 cells contained numerous well defined stress fibers that protruded into the cell interior and formed numerous focal adhesions. These features readily differentiated MDA-MB-435 cells from the other breast cancer cells. It also appears that MDA-MB-435 focal adhesions were signaling effectively as evident with the correlated transient increases in pFAK, pSrc(Y416) and pERK following PMA treatment (Figure 4), and in the adhesion-induced activation of pFAK and pMEK (Figure 7).

The integrin co-receptors, uPAR and VEGFR, play important roles in the progression of cancers [11,14]. All the breast cancer cell lines and Hek-293 cells expressed uPAR but only MCF7 cells expressed high levels of VEGFR. The expression of uPAR by all the cancer lines, is in keeping with uPA/uPAR being a prognostic marker of breast cancer. uPAR participates in many cellular processes by interacting with β_1 _and β_3 _integrins and modulate their signaling, by serving as a binding site for VN and by inducing cytoskeletal reorganization [14,30]. The delivery of an adequate supply of blood to malignant tumors is required for their rapid expansion as they must receive nutrients and oxygen imposed by tumor growth [11]. Many cancers meet their blood supply demands by inducing angiogenesis, and there is increasing evidence implicating integrin signaling, generated by interactions with ECM proteins and with VEGFR, as a major modulator of cancer-induced angiogenesis [4,11]. The high expression of VEGFR by the non-metastatic MCF7 cells, may indicate a critical role for angiogenesis in the progression of MCF7 breast cancers. In MDA-MB-435 and MDA-MB-231 metastatic tumors, uPAR-mediated degradation and remodeling of the ECM to facilitate metastasis [14], is likely of more importance than VEGFR-mediated angiogenesis in the progression of these cancers.

Breast carcinomas have been reported to contain higher MAPK activity than benign breast tissue, and there is a positive correlation between ERK activation and shorter relapse-free survival period [31,32]. Other studies reported a positive correlation between ERK activation and a less aggressive disease and better survival rates [33]. The magnitude and temporal organization of ERK activity also correlates with specific biological responses [34,35]. In intestinal cells, transient ERK activity results in cell growth, while a strong and sustained ERK activity leads to cell cycle arrest [35]. In our study, we identified marked differences in the regulation of MAPK signaling and ERK activation within the cancer lines. The levels of pMEK and pERK in adhered MDA-MB-435 and MCF7 cells were transient, reaching a maximum within two hours of PMA treatment, while pMEK levels in MDA-MB-231 cells remained constitutively high and pERK levels continued to increase. Furthermore, in contrast to MDA-MB-231 cells in which pMEK levels were adhesion-independent and pERK levels were adhesion-dependent, pMEK levels were adhesion-dependent and pERK levels were adhesion-independent in MDA-MB-435 cells. We speculate that differences in the activity of phosphatases within the cell lines accounted for the different pERK levels, and that alterations in the regulation of phosphatase activity between various breast cancers contributes to variations in their phenotypes. Furthermore, our data supports a relationship between pERK and the metastatic capacity of the cells, as adhered metastatic MDA-MB-435 and MDA-MB-231 cells contained elevated pERK levels compared to non-metastatic MCF7 and Hek-293 cells (Figure 7B).

The autophosphorylation of FAK at Y397, serves as binding site for Src-family protein kinases which following further activation, phosphorylates a variety of substrates such as paxillin, and activates a number of protein kinase cascades [12,36]. The expression of Src correlates with metastatic activity of breast cancers, and integrin signaling through Src can be FAK-mediated or FAK-independent as Src in cancers expressing β_3 _integrins [13,37]. In our studies, all proliferating cells expressed activated pSrc(Y416) but only metastatic MDA-MB-435 cells showed an induction of pSrc levels following PMA stimulation. As this was the only breast cancer to express α_v_β_3_, we believe that FAK-independent activation of Src by α_v_β_3 _contributes to the metastatic phenotype of MDA-MB-435 breast cancers.

The ability of metastatic cells to loosen their adhesion to the ECM and acquire a migratory phenotype that enables the cancer to move through and expand into other tissues are processes regulated by FAK-Src signaling [36]. High FAK expression occurs in cancers, including breast cancers, and FAK expression is correlated with a highly malignant and metastatic phenotype [38-40]. Our own observations are consistent with these previous studies, with the breast cancers containing higher levels of FAK than Hek-293 cells. In addition, pFAK levels were markedly elevated in MDA-MB-231 cells, which may reflect the invasive phenotype of this cancer [15]. The higher levels of pFAK in MDA-MB-231 may contribute to focal adhesion turnover and reorganization, resulting in fewer stable focal adhesions and fewer contacts between integrins and actin stress fibers. This speculation is supported by our observation that MDA-MB-231 cells formed the fewest focal adhesions of the three breast cancers, which may allow for them to more readily disengage from the ECM. Their capacity to remodel and degrade ECM, partially using uPAR-mediated processes, would then facilitate their migration and invasion into other tissues. Other studies have demonstrated that FAK-mediated signaling to ERK does not follow a single linear pathway [36]. FAK enhances the phosphorylation of MEK1 at Ser-298 facilitating ERK2 activation [41]. Thus, FAK signaling can potentially affect the tumorogenic, metastatic, and invasiveness of breast cancers by modulating Src and MAPK signaling.

## Conclusion

Our study identifies that there is heterogeneity in integrin expression, integrin cellular structures, integrin co-receptor expression and integrin signaling within breast cancers. This heterogeneity likely contributes to the phenotypic heterogeneity of breast cancer. More studies are needed to better define the role of integrin-associated structures in regulating integrin signaling and the role of integrin signaling in breast cancer metastasis and invasiveness. Our data also underscores the need for better categorization of breast cancers into smaller groups to allow for more efficacious therapeutic treatment.

## List of abbreviations

BSA: bovine serum albumin; ErbB2: epidermal growth factor receptor 2; ECM: extracellular matrix; Fg: fibrinogen; FN: fibronectin; F-actin: Filamentous actin; FAK: focal adhesion kinase; PMA: phorbol; 2-myristate 13-acetate; uPAR: urokinase receptor; uPA: urokinase plasminogen activator; VEGFR: vascular endothelial cell growth factor receptor; VN: vitronectin.

## Competing interests

The authors declare that they have no competing interests.

## Authors' contributions

The study was designed by AT, XL, YL and TAH. All authors have read and approved the final manuscript. TH ensured funding. AT performed data collection, statistical analyses and interpretation of the cell adhesion, PMA treatment and adhesion-induced signaling results. AT collected integrin expression data by western analysis and wrote first draft of manuscript. XL collected integrin expression data by immunocytochemistry analysis and performed data collection, statistical analyses and interpretation of the immunocytochemistry results. YL performed FACS integrin data collection, statistical analyses and interpreted the results. YL also collected pFAK data for Figure 3 and data for Figure 5. TH wrote all manuscript revisions, designed most experimental approaches taken, and performed statistical analyses and interpretation of all the results.

All authors have read and approved the final manuscript.

## Pre-publication history

The pre-publication history for this paper can be accessed here:

http://www.biomedcentral.com/1471-2407/11/293/prepub
